# Occupational pesticide intoxications among farmers in Bolivia: a cross-sectional study

**DOI:** 10.1186/1476-069X-5-10

**Published:** 2006-04-21

**Authors:** Erik Jørs, Rafael Cervantes Morant, Guido Condarco Aguilar, Omar Huici, Flemming Lander, Jesper Bælum, Flemming Konradsen

**Affiliations:** 1 Department of Occupational and Environmental Medicine, Odense University Hospital, Denmark; 2 CARE, La Paz, Bolivia; 3 Instituto Nacional de Medicina Ocupacional(INSO), La Paz, Bolivia; 4 Department of Occupational and Environmental Medicine, Skive Hospital, Denmark; 5 Department of International Health, Institute of Public Health, University of Copenhagen, Denmark

## Abstract

**Background:**

Pesticide use and its consequences are of concern in Bolivia due to an intensive and increasing use.

**Methods:**

To assess the magnitude and reasons for occupational pesticide intoxication, a cross-sectional study with interviews and blood-tests was performed among 201 volunteer farmers from 48 villages in the temperate and subtropical valleys in the eastern part of the Andes Mountains in Bolivia. Of these 171 male farmers using pesticides in their agricultural production were used in the statistical analysis, including linear- and logistic regression analysis.

**Results:**

This study documented a frequent use of the most toxic pesticides among farmers who have had almost no instructions in how to use pesticides and protect themselves against the dangers of intoxication, reflected in the hazardous practices used when handling pesticides. Symptoms of intoxications were common in connection with spraying operations. The risk of experiencing symptoms and the serum cholinesterase activity were influenced by whether or not organophosphates were used and the number of times sprayed. The experience of symptoms was moreover influenced by the hygienic and personal protective measures taken during spraying operations while this had no influence on the serum cholinesterase level.

**Conclusion:**

The study showed that occupational pesticide intoxications were common among farmers and did depend on multiple factors. Pesticide use is probably one of the largest toxicological problems in Bolivia, and a coordinated action by authorities, society and international bodies is needed to limit the number of intoxications and the environmental pollution.

## Background

In Bolivia almost half of the population of 8.3 million is living on farming and related activities, contributing to 15% of the Gross National Product. The agricultural sector can be divided into two categories, one which is cash crop producing, mechanized large farms in the tropical Amazon lowlands, and the other which is small-scale farming in the subtropical valleys of the eastern slopes of the Andes Mountains and on the temperate plateau – 'the Altiplano' – 4,000 m above sea level.

In most low income countries, intensification of agriculture and to a lesser extent the public health control of vector borne diseases have lead to an increase in the use of pesticides. In Bolivia the value of the imported pesticides has increased 20% per year during the last decade, which is substantial even compared with other low-income countries (FAOSTAT data, 2004).

It is vital that the impact of this increasing use of pesticides [1] can be assessed and the information brought forward to guide governments and international bodies in the formulation of appropriate policies and to evaluate current initiatives. This documentation is lacking in Bolivia as in most other low income countries.

The studies conducted in Bolivia during the past decades have shown insufficient mechanisms to control and regulate pesticide imports and sales, lack of knowledge about handling the pesticides, rudimentary use of personal protective equipment and insufficient protective hygienic measures applied among farmers [2-5]. Likewise, frequent experiences of acute intoxications among farmers when handling pesticides, easy access to pesticides leading to cases of self-inflicted intoxications in the population and pesticide residues above recommended levels in foodstuff are reported [2-5].

This study focuses on the assessment of occupational pesticide intoxications and risk factors for these among the farmers in the valleys of the eastern slopes of the Andes Mountains in La Paz County, Bolivia.

## Methods

### The study area and background

The study was done as part of the Plag-Bol project, the objective of which is to lower the number of intoxications and reduce the environmental pollution from pesticides. The project activities include education of health personnel in diagnosis, treatment and prevention of intoxications, the promotion of Integrated Pest Management strategies (IPM) among farmers, and a general awareness raising concerning the possible dangers for health and environment from pesticides among the public through information spread by mass-media and educational institutions.

The data presented were gathered over a four week period in March and April 2002 from 201 farmers living in 48 small villages with a total population of approximately 10,000 people. Of these, approximately 2,000 are male farmers, and our sample then represents about 10% of the male farmers, and 2% of the total population in the villages. Due to the mountainous terrain, the climate in the study area varies from temperate to subtropical making it possible to grow a wide variety of crops such as vegetables, corn, potatoes, flowers, fruits, coffee and rice, which are most often marketed in the nearby capital, La Paz. The spraying season is from October to May, although some spray throughout the year, especially the farmers growing tomatoes and flowers, crops which can be harvested several times a year.

The study was approved by the Medical Ethical Committee in Bolivia and the Bolivian National Institute of Occupational Health (INSO) and was in compliance with the Helsinki Declaration.

### Design

The farmers participating in the study were from the villages where the Plag-Bol project was taking place, and the data were collected before awareness raising or any other activity took place in the project. The participating villages were selected after consultations with the local farmers' representatives. They were known to be villages with significant use of pesticides and a good accessibility by road or river thus facilitating later project intervention activities. Farmers were invited to village meetings, where they were briefed about the study, its relevance and what health dangers the blood tests could pose. They were asked to volunteer for the study and then interviews and tests were carried out on 201 farmers of which 19 were females. They had a mean age of 36 years (range 15–79), had been working for 20 years in agriculture (range 1–60), and cultivated on average 1.6 hectares of land (range 0–11). All participants signed an informed consent form before the interviews were conducted and the blood samples were taken.

The interviews and blood tests were used to evaluate a possible influence on the health of the farmers by pesticide use and to identify risk factors for intoxication. A maximal number of persons of 250 was estimated as realistic for the statistic purpose of the investigation based on knowledge of symptom frequency and cholinesterase measurements from a former study [3]; for a 25% fraction in the smallest exposure group and a 20% symptom score this gave an 80% power of detecting an odds-ratio of 2.4, while a difference of 0.7 IU of cholinesterase could be detected with the same power. These figures were thought as relevant minimal detectable differences.

Interview forms used in Bolivia, Denmark and the US were the basis for a questionnaire consisting of closed and open-ended questions, including i) age, sex, education, family status, the suffering from any diseases, smoking habits etc., ii) the size of cultivated land, crops grown, pest affecting the different crops and the way to deal with them; iii) knowledge, attitudes and practice when buying, handling and storing pesticides; and iv) perceived health impact, perceived dangers of pesticides, experiences with acute pesticide poisonings and toxic symptoms in connection with spraying. When symptoms were assessed the interviewed was asked if he had felt ill in connection with spraying during the past year, and if the answer was yes, he was asked to specify, which symptoms he had experienced. The interviewer could mark symptoms on a pre-elaborated list or add symptoms if they were not on this list.

The questionnaire was pilot tested and adjusted when necessary and the survey was conducted by trained health professionals and agronomists in order to control inter-observer variability.

The blood tests were taken by the laboratory personnel from the National Institute of Occupational Health in La Paz at the time of the interviews; the participants signed an informed consent before blood-tests were taken. The tests were centrifuged on site, the serum frozen and transported for analysis of serum cholinesterase activity (ChE) at the laboratory at Odense University Hospital, Denmark. The ChE activity was measured by a spectrophotometric method where ChE activity is used in the first step of a reduction of potassium hexacyanoferrate leading to a color change that can be measured with a variance below 2.3% within the same set of analysis. The measurements were given in kilo units per liter (kU/L) [6]. The ChE activity is known to be lowered by intoxication with organophosphates and carbamate pesticides and to be influenced by weight, sex, age, liver-diseases and the use of contraceptive pills [6]. Based on the interviews with the farmers the WHO toxicology classification was used to identify and classify the different pesticides mentioned [7].

### Data analysis

Of the 201 farmers interviewed, 186 farmers used pesticides, of which 171 were males. In the analysis of occupational risk factors for a depressed ChE activity this group of 171 farmers was used, excluding one with a missing blood test. One hundred and fourteen of the 171 farmers had been spraying within a month prior to the interview, and this group of 114 male farmers was used to test risk factors for the experience of symptoms in connection with spraying (symptoms during or immediately after a spraying operation).

The first group of risk factors tested for was the number of times sprayed in the past month and the use of organophosphates (OPs) or not during the past month. These two variables were aggregated into one coded 0 = no spraying, 1 = spraying only pesticides other than OPs, 2 = spraying 1–3 times with OPs, 3 = spraying >3 times with OPs. It was assumed that the group with the heaviest exposure to pesticides would be the group having sprayed more than three times with OPs, and that this would be reflected in the experience of symptoms and in the blood test.

The second group of factors tested was the protective behaviors performed when spraying. They were tested one by one and in an aggregated variable including the use of personal protective equipment (using plastic poncho, mask, gloves or boots while spraying), the level of personal hygiene measures (changing clothes, washing hands, washing body after spraying; refraining from eating/chewing coca leaves while spraying), avoiding re-entry into a newly sprayed field, refraining from blowing/sucking the nozzle of the knapsack sprayer when cleaning it and reading instructions on the pesticide container before use. The aggregated variable was expressed as a score where each protective behavior counted 1 point if performed. The participants were divided into four groups of appropriated size, expressing the number of protective behaviors they performed when handling pesticides (0–3, 4–5, 6–7 and >7).

The possible confounders as age, body mass index (BMI), smoking, years of farming and educational level were analysed one by one and all together. Women were excluded because of a known influence of sex on ChE activity and owing to the few women participating in the study. Data of alcohol use was not included in the questionnaire. Alcoholism (a daily intake of alcohol) could be a confounding factor, but profound knowledge from these areas tells us that alcohol consumption on a daily level is almost unknown for economic and traditional reasons. We did however ask for alcohol consumption during the last 24 hours prior to the blood tests were taken, and found no reason for excluding any of the farmers due to this.

Data were entered and analyzed in the statistical program STATA 8.0. Frequency analysis, χ 2-test, t-test, non-parametric test, linear regression and logistic regression were used in the analysis.

## Results

### Pesticides used

The ten most common pesticides used by the farmers, according to the WHO classification [7], are listed in Table 1. Insecticides were used by 97% of the farmers (mainly organophosphates 88%, pyretroides 48%), followed by fungicides (63%) and herbicides (31%). Aldrin, dimethoate and parathion were used, though not allowed to be imported and restricted or banned through international treaties signed by Bolivia.

**Table 1:** Classification of pesticides used by farmers, the ten most used active ingredients and their characteristics (n = 171)

**Active Ingredient**	**Used by percent of farmers**	Toxicological classes*	**Chemical class**	**Classification by main use**
Methamidophos	69 %	Ib	Organophosphate	Insecticide
Sulphur	40 %	U		Fungicide
Propenophos	34 %	II	Organophosphate	Insecticide
Cypermethrim	26 %	II	Pyretroide	Insecticide
Spinosad	25 %	U		Insecticide
Propineb	25 %	U		Herbicide
Parathion	23 %	Ia	Organophosphate	Insecticide
Dimethoate	16 %	II	Organophosphate	Insecticide
Permethrin	15 %	II	Pyretroide	Insecticide
Lambda cyhalotrin	11 %	II	Pyretroide	Insecticide

* Ia extremely hazardous, Ib highly hazardous, II moderately hazardous, III slightly hazardous, U active ingredient unlikely to present any harm in normal use, O obsolete (WHO classification).

### Pesticide handling

The level of knowledge among the farmers is seen from Table 2, where answers about factors with a possible influence on intoxication of humans and pollution of the environment when handling pesticides are listed.

**Table 2:** Factors of importance for intoxications in humans and pollution of environment when handling pesticides (n = 171)

**Factor**	**% positive answers**
Using gloves when handling pesticides	16 %
Using boots when handling pesticides	16 %
Using a plastic poncho when handling pesticides	3 %
Using a mask when handling pesticides	17 %
Washing hands after handling pesticides	69 %
Washing the whole body after handling pesticides	54 %
Changing clothes after handling pesticides	47 %
Chewing coca, smoking or eating during a spraying session	15 %
Spraying less than one day before harvest	25 %
Spraying products after harvesting and before taking them to the market	19 %
Entering a field the same day is sprayed	27 %
Blowing or sucking the nozzle of the knapsack sprayer when obstructed	49 %
Mixing pesticides at the borders of rivers or ponds	35 %
Washing knapsack sprayer in or at the borders of rivers or ponds	30 %
Throwing empty pesticide containers in the fields or into the rivers	72 %
Using pesticides as medicine for skin infections in humans (mainly scabies)	16 %
Keeping pesticides locked up	8 %

Twenty five percent of the farmers had received some instructions on how to use pesticides, mainly from salesmen; and seventy four percent told that they did read the instructions on the pesticide containers before use. However, the meaning of the color marked on the pesticide containers signalizing the toxic potential of that specific pesticide was unknown to seventy one percent of the farmers.

### Symptoms and risk factors

**Figure 1:**
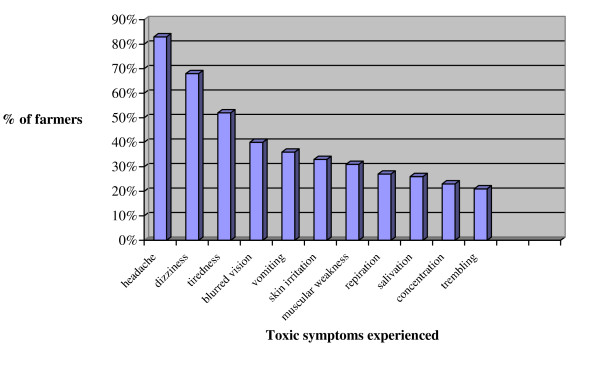
Symptoms experienced by farmers in connection with spraying pesticides within the last year (n = 128)

Seventy percent of the male farmers using pesticides reported having experienced symptoms of intoxication in connection with one or more spraying sessions during the last year, while forty five percent of those who have been spraying past month did experience symptoms. The most frequent symptoms mentioned were headache, dizziness, tiredness, blurred vision and vomiting, Figure 1.

In an aggregated variable, expressing the number of times sprayed and whether or not OPs were used, the experience of symptoms in connection with spraying was found to be depending on the degree of pesticide exposure as seen in Table 3. When comparing the experiences of toxic symptoms among those who had sprayed >3 times in the previous months with those who had sprayed 3 times or less an OR of 3.58 (95% CI 1.44-8.92) was found after controlling for the number of protective behaviors practiced. Analyzing for the use of OPs or pesticides other than OPs and controlling for the number of protective behaviors practiced, an OR of 2.96 (95% CI 0.96-9.12) for having symptoms after spraying with OPs was found.

**Table 3:** Odds Ratio (OR) for having experienced symptoms of acute pesticide poisoning after spraying past monthaccording to exposure status among male farmers (n = 114)

	%	Unadjusted		Adjusted*	
		
		OR	95% CI	OR	95% CI
Sprayed only pesticides other than organophosphates (OPs) past month	22	1(ref)	-	1(ref)	-
Sprayed from 1–3 times with OPs past month	45	2.04	0.70 – 5.99	1.91	0.58 – 6.30
Sprayed more than 3 times with OPs past month	33	6.09	1.96 – 18.97	5.97	1.63 – 21.96
					
>7 precautions taken when handling pesticides	17	1(ref)	-	1(ref)	-
6–7 precautions taken when handling pesticides	33	5.63	1.37 – 23.06	5.15	1.17 – 22.67
4–5 precautions taken when handling pesticides	32	4.17	1.01 – 17.18	5.19	1.15 – 23.42
0–3 precautions taken when handling pesticides	18	10.83	2.25 – 52.20	13.88	2.60 – 74.11

Logistic regression analysis, * the OR were mutually adjusted.

The number of protective behaviors performed while handling pesticides also showed an influence on the risk of experiencing symptoms after spraying – the more protective behaviors performed the less chance of experiencing toxic symptoms after spraying, as can be seen from Table 2. When analyzing the protective behaviors one by one, controlling for the type of pesticide used and the number of times sprayed, 'no use of gloves' (OR 2.87, 95% CI 0.90–9.11), 'no use of a mask' (OR 2.72, 95% CI 0.96–7.73), 'the habit of blowing/sucking the nozzle of the knapsack sprayer when obstructed' (OR 4.00, 95% CI 1.70–9.45) and 'not reading the instructions on the container before using the pesticide' (OR 3.24, 95% CI 1.19–8.87) showed elevated OR for the experience of symptoms and seemed to have greater importance for the experience of symptoms than the rest of the assumed protective behaviors.

Possible confounders like age, BMI, years of farming and educational level were not shown to have any significant influence when taken into the analysis. Smoking had, but as it only increased OR without affecting the significance of the analysis, and resulted in some very broad confidence intervals due to the few smokers and the limited size of the study, it was not included in the analysis. The educational level (being an analphabet, up to six years of public school, 6–10 years of public school, having a technical or a higher education) was shown to have an influence on the number of protective measures realized when spraying, (p = 0.04, Spearmann rank correlation test).

### Cholinesterase activity and risk factors

In the aggregated variable, expressing the number of times sprayed and whether or not OPs were used, the mean ChE activity was found to be depending on the degree of pesticide exposure as seen in Table 4.

**Table 4:** Serum cholinesterase activity according to exposure status past month and Body Mass Index among farmers (n = 170)

	%	Mean ChE activity kU/L	Unadjusted		Adjusted^*^	
			
			Coefficient	95% CI	Coefficient	95% CI
Not having sprayed during the last month	22	8.36	-	-	-	-
Sprayed only with pesticides other than OPs	17	7.60	-0.76	-1.46 to -0.06	-0.86	-1.64 to -0.09
Sprayed from 1–3 times with OPs	34	7.34	-1.02	-1.62 to -0.43	-1.19	-1.84 to -0.53
Sprayed more than 3 times with OPs	27	6.84	-1.56	-2.15 to -0.90	-1.62	-2.31 to -0.92
Constant			8.36	7.90 – 8.82		
						
>7 precautions taken when handling pesticides	18	7.53	-	-	-	-
6–7 precautions taken when handling pesticides	30	7.47	-0.07	-0.82 – 0.68	-0.06	-0.78 – 0.66
4–5 precautions taken when handling pesticides	33	7.51	-0.02	-0.76 – 0.72	-0.22	-0.92 – 0.48
0–3 precautions taken when handling pesticides	19	7.45	-0.08	-0.90 – 0.74	-0.08	-0.85 – 0.69
Constant			7.53	6.93 – 8.13		
						
BMI>25	73	8.00	-	-	-	-
BMI≤ 25	27	7.23	-0.77	-1.26 to -0.27	-0.49	-1.0 – 0.02
Constant			8.0	7.58 – 8.43		
						
Constant					8.97	8.13 – 9.80

Linear regression analysis, *the regression coefficients were mutually adjusted.

Analyzing the number of times sprayed, controlling for the number of protective behaviors performed and BMI, a ChE activity of 8.36 kU/L for those who have not being spraying was found, compared to a ChE activity of 7.60 kU/L for those who have being spraying from 1–3 times (p = 0.03) and a ChE activity of 7.12 kU/L for those who have being spraying >3 times (p < 0.01).

Comparing the group who has been spraying with OPs with the group who has not, controlling for the number of protective behaviors performed and the BMI, a mean ChE activity of 7.11 kU/L for those who have sprayed with OPs compared to a mean ChE of 8.03 kU/L for those who have not, was found (p < 0.01).

The number of protective behaviors did not influence the ChE activity significantly. The only significant protective behavior was reading instructions on the pesticide container before use or not, ChE activity 7.46 kU/L versus 6.84 kU/L (p = 0.02), and controlling for whether or not OPs were used, the number of times sprayed, and BMI.

BMI was shown to be a confounder of the ChE activity and was taken into the analysis, but other potential confounders like age, smoking, years of farming and education showed no effect on results and were not included in the analysis presented in Table 4.

The mean ChE activity among those with symptoms after spraying past month (n = 51) was 7.07 kU/L compared to a mean ChE activity of 7.46 kU/L among those without symptoms after spraying (n = 63) (p = 0.14).

## Discussion

This study documented the use of very toxic pesticides among farmers. The farmers had received almost no instructions about the dangers of pesticides and preventive measures to protect themselves and the environment leading to very hazardous practices when handling pesticides. Possible symptoms of intoxications and a depressed ChE activity after spraying sessions seemed to be common and were related to spraying intensity, spraying with OPs or not and the number of protective behaviors performed when handling pesticides.

The situation where more than seventy five percent of the farmers used pesticides either not registered for use in Bolivia or restricted by international conventions signed by Bolivia needs attention [9-13]. The reasons might be their free availability owing to smuggling and the control measures regarding import and sales of pesticides not being enforced [2,3,14]. Pesticides of all kind are sold to everyone on the street and in shops, where the salesmen mostly operate without a license and do not comply with the Bolivian law regulating the sale and marketing of pesticides [2,3]. Pesticides are often kept next to foodstuff and only to a limited extent locked up in a safe place. The result is frequent intoxications, not only in occupational circumstances but also due to accidents and self-harm. From the Plag-Bol study it was reported, based on review of hospital registers and interviews with farmers, that pesticides are by far the most common agent for suicidal attempts and that ninety two percent of the fatal intoxications with pesticides were self-inflicted [15].

To restrict the accessibility, pesticides should be kept locked up, license to pesticide dealers should be controlled, farmers could be licensed allowing only persons with license to buy and use pesticides, and a positive list with a restricted number of pesticides excluding the most toxic ones could be established as suggested by some authors [16,17]. This would have an effect not only on occupational intoxications, but also suicidal and accidental intoxications would be minimized [18]. Studies have shown that by applying alternative and ecologically based methods, pesticide use can be decreased by at least fifty percent without reducing the yield [19,20], and this might be one of the possibilities for controlling this increasing prevalence of pesticide poisonings.

The frequency of self reported work related symptoms of pesticide poisonings was higher than found in previous Bolivian studies from 1989 and 2000, which showed a lifetime experience of poisonings of 10.5% and 48% respectively [3,4]. A study from Nicaragua reported a frequency of 11% of responding farmers having experienced symptoms of intoxication after spraying during the last month, 25% in the last 12 months and 48% at one point in time [8]. The variation between the studies might be due to differences in crops cultivated, pest pressure, spraying intensities and toxicity of the pesticides used. Recall-bias due to different recall periods applied might be another explanation. The difference between the Bolivian studies might also reflect the significant increase in the use of pesticides in Bolivian agriculture over the last decade.

The knowledge of how to handle pesticides and the use of protective measures were poor in the actual study, as seen from Table 2, and also found in earlier studies from Bolivia and other low-income countries [2,3,21-23]. One possible explanation could be the lack of access to information, and a general, low level of education leaving many as functional illiterates. Although seventy five percent of the farmers reported reading the information on the pesticide containers, clearly, they did not understand the information on the label or they only read information that enabled them to apply it more efficiently, and not for safety reasons. The limited use of personal protective equipment might be due to the lack of availability, lack of money to buy or the inappropriateness of protective measures when used in hot climates as found in other studies [24,25], and pointed out by farmers to the Plag-Bol project (personal communication).

The significant 'dose – response' associations as seen in this study between the number of times sprayed/the use of OPs or not/the number of protective behaviors realized while handling pesticides on one hand and the experience of symptoms of intoxication and the finding of changes in ChE activities are also found in other studies from low-income countries, where the use of some personal protective equipment, a certain level of personal hygiene when spraying, and knowledge of pesticide dangers have been shown to prevent toxic symptoms and/or a depressed ChE activity [1,21-23,26]. Some studies do not find the relationship between protective measures undertaken and symptoms of intoxication [19,27], probably reflecting the difficulty in analyzing a single occupational risk factor, without taking other closely related factors into account at the same time. Therefore it might be sounder to aggregate various closely related protective factors into a score as we did in the actual analysis. One might argue that this takes away the idea of identifying specific risk factors to be targeted in an intervention, but only targeting e.g. one risk factor like 'reading the label on the pesticide containers before use' make little sense, if you do not target other important risk factors like personal hygiene measures, the use of personal protective equipment, reentry practices etc., as they all might influence the risk of having an intoxication.

The associations of symptoms and ChE levels with a higher frequency of pesticide use could reflect a cumulative effect of repeated exposure, but it could also be explained simply by the fact that people who have used pesticides more often have had more opportunity to develop acute symptoms and/or a lowered ChE level.

The lack of association between the number of protective measures taken during spraying operations and the ChE activity could be due to a too large interval between exposure and sampling of the blood tests, as the level of ChE activity returns to normal within days to weeks after exposure to organophosphates, and can thus only serve as a measure for fairly recent exposures [6]. Information bias might also explain this lack of association, if people claim to have realized protective behaviors without really having done it.

The time interval between spraying and the blood test taken might also explain the lack of significant correlation between symptoms and serum ChE levels, although the farmers without symptoms did have a higher mean level. A better indicator would have been red blood cell cholinesterase activity as a marker of biological effect, whereas serum ChE is a marker of exposure.

A limitation of the study is the lack of possibility to differentiate between the seriousness of the intoxications experienced within the last month, as we did not ask for the number and seriousness of the symptoms experienced. Neither do we have data from a medical examination, as farmers mostly do not seek treatment for these normally less serious intoxications with symptoms lasting for only hours to a day. The symptoms mentioned by the farmers like headache, dizziness, tiredness, blurred vision, vomiting, salivation and muscular weakness are not specific and might, in some of the cases, be due to other causes than pesticide poisoning. Another limitation is the non-random selection using volunteers attending a meeting in the villages. This may decrease the ability of the study to generalize the findings to other regions, but should however not hamper the validity of the data. Years of experience of working among Bolivian farmers, indicate to the authors that the group participating in this study seemed to be quite typical of the small-scale farmers from these areas.

Due to inter- and intra-individual variance the ChE values must be interpreted with caution, and a normal variation of ChE for a population is often claimed to be too broad for any practical use, whereas the interpretation of individual values demands at least two measures to be taken, where one 0-value must be taken when the person has not been exposed to pesticides for some time. On a group level in an epidemiological study, we think however that it is possible to compare the mean serum ChE activity of different groups with different exposure circumstances, assuming that individuals with different basis activity of ChE are evenly distributed within the groups [6].

## Conclusion

The study showed that occupational pesticide intoxications were common among farmers and were related to the frequency of spraying, the use of organophosphates and the number of protective measures undertaken by the farmers when spraying. Pesticides of the most toxic classes were widely sold and used, also those banned or restricted by international conventions and laws. The farmers had very little knowledge about the dangers of pesticides and the benefit of protective measures when handling pesticides.

As the use of pesticides probably is one of the most important toxicological problems in Bolivia, a coordinated action by authorities, society and international bodies, including pesticide producing countries, is urgently needed to be able to limit the number of intoxications and pollution of the environment.

## Competing interests

The author(s) declare that they have no competing interests.

## Authors' contributions

EJ was the leader of the research. He contributed to all phases in the research project from conception and design, the acquisition and analysis of data and the writing of the manuscript.

RCM contributed to the conception and design and the acquisition of data.

GCA contributed to the conception and design and the acquisition of data.

OH contributed to the conception and design and the acquisition of data.

FL contributed to the conception and design, the acquisition of data and the revising of the manuscript for intellectual content.

JB contributed to the design, the analysis of data and the revising of the manuscript for intellectual content.

FK contributed to the analysis of data and the revising of the manuscript for intellectual content.

All authors have given their final approval of the version to be published.
